# A General Survey of Developments during the Year 1915

**Published:** 1916-01-08

**Authors:** 


### NATIONAL INSURANCE ACT DEVELOPMENTS.

### A General Survey of Developments during the Year 1915.

THE amount of general public interest that has been taken in National Health Insurance matters during the year 1915, as indicated by notice in the Press, has been comparatively slight as contrasted with recent years. This does not mean, however, that the developments of the system have been either few or insignificant. The lack of interest is attributable to the more absorbing interest that has been taken in matters directly relating to the war.

It is somewhat remarkable, however, that so little public interest should have been taken in the developments that have been made during the year in view of the fact that such developments have been rendered necessary, almost without exception, by the abnormal conditions due to the war. Normal developments, such as the first valuations, have had to be postponed; but, on the other hand, various special problems have arisen, and these have demanded attention from those who are responsible for the administration of the Acts. We have from time to time drawn attention in THE HOSPITAL to the most important changes that have occurred during the year, and the present is a fitting opportunity for a general survey of the developments that have been made in the first complete year under war conditions.

### THE AMENDMENT ACT.

In a retrospect of this character the first item to be noted is the fact that it became necessary in March last to place a further short Amendment Act upon the Statute Book. This was essentially an Act due to the war, for it was almost exclusively concerned with the insurance of men in the Naval or Military Service of the Crown. It will doubtless be remembered that the principal provision of the Act gives relief to approved societies to the extent of 5s. a week—thereby covering the payment of disablement benefit—in respect of sailors or soldiers who, upon their discharge, are granted a *total* disablement pension.

This relief was a recognition that the cost of providing for those injured in the war is a national charge, to be met out of public funds, and not from the funds of approved societies. It has recently come to our notice, however, that up to the present very few *total* disablement pensions have been awarded, while on the other hand a large number of *partial* disablement pensions of as much as 22s. 6d. a week have been given to men whose injuries preclude them from obtaining employment. As these men are still members of their societies and incapable of work they are entitled to sickness benefit up to the maximum of twenty-six weeks, and thereafter to disablement benefit at the rate of 5s. a week so long as the incapacity for work continues. In some cases we have heard of there is apparently no prospect of recovery within the year, and, as the grant of the pension will not be reviewed until the year has expired, it is clear that the society will have had to pay a sum of £13 from which it would have been relieved if the pension had been granted as a *total* disablement allowance. Moreover, throughout the whole of the period the member would, in the circumstances mentioned, have been in receipt of a larger weekly allowance, taking into account both sources of income, than if the pension had been given at once as a total disablement allowance of 25s. a week. It is therefore clear that the Amendment Act of 1915 has not yet furnished the relief that the societies expected and have the right to expect.

In some other respects the provisions of the Act of 1915 have been particularly beneficial. For instance, approved societies were empowered to pay benefits pending the decision of the Admiralty or the Army Council with regard to the grant of pension, and to recover the amounts so paid when the decision as to the pension had been settled. In view of the delays, which appear to be inevitable, in settling claims for pension, the provision whereby the approved societies have been able to afford immediate relief has been of much value to the disabled soldiers, who would otherwise have been temporarily destitute.

### THE ENLISTMENT OF INSURED PERSONS.

From the point of view of the approved societies the principal features of the year are undoubtedly the large transference of members from the employed contributor's class to the Army class, and the large influx of female members entering insurance for the first time.

Owing to the large number of enlistments there was at one time serious apprehension on the part of approved societies as to the adequacy of the allowance from contributions for administration expenses. By increasing the amount of the normal allowance for Army members, and providing further for a special grant for the half-years in which transfer is made to or from the Army class, the Insurance Commissioners have set at rest the apprehensions on this score. Another development directly due to the large number of enlistments of insured persons is the decision to dispense with insurance cards and stamps in respect of insured persons in the Army. In place of the insurance cards a system of direct accounting in bulk has been inaugurated. It is too early yet to pronounce definitely upon the efficacy of the plan, but it is hoped that it will effect economy both in time and material, and will thus be the means of saving money. If the experiment prove successful, it should be watched very closely, with a view to the possible elimination of insurance cards and stamps for the general body of insured persons in future.

### WOMEN ENTERING EMPLOYMENT BECOME INSURED.

The increase in the number of women who are insured is a result of the employment of women in the place of men on active service. Viewed at first with some misgivings as to the possible adverse effect of this large influx of female lives upon the finances of approved societies, it now seems to be generally admitted that the immediate effect has been to reduce substantially the rate of sickness claim in women's societies, and whatever may be the ultimate result, the actuarial advisers to the Commission appear to be content to allow the present scales of benefit and contribution to remain unaltered for the time being.

### The Demand for Economy.

There has been an insistent demand in Parliament, and elsewhere, that steps should be taken to ensure economy in the administration of National Insurance. Mr. Charles Roberts, who is now in charge of these matters in the House, indicated that the only possible economies that could be made were in regard to detail. A considerable sum should already have been saved in printing, for the number of circulars and Orders has been substantially reduced. Saving of this nature, though important, frequently leaves out of account the legitimate desire for information. Thus, for instance, there has not appeared during the year the report on the year's proceedings as for the two previous years. This naturally makes it impossible to compare readily the progress that has been made, but it is to be hoped that the omission to publish an annual report will be temporary only, and that in due course, after the war, a report covering the period since the last one was rendered will be prepared and published.

### Simplification of Detail. The Arrears Scheme.

The question of economy is largely concerned with the simplification of administrative detail. Dispensing with Army cards is a case in point. There is, however, still much scope for the simplification of the present National Insurance system. During the year the first real experience has been gained of the arrears regulations which were designed as an improvement upon the original plan, and it can be said at once that the amount of detail involved in the practical application of the revised plan has been so great as to call forth insistent demands from all descriptions of approved societies for simplification. We put forward suggestions ourselves in our issue for August 14 last, which, if adopted, would have the effect of reducing detail work to a minimum, the underlying idea being that there is not the same necessity for penalising arrears of contribution when the payment of contributions is compulsory as when the payment is voluntary. All that is required is, in our opinion, a definite standard whereby it may be possible for approved societies to decide whether a member has ceased to be normally engaged in employment within the meaning of the Act.

### Medical and Sanatorium Benefits.

Another feature of the year as affecting National Insurance matters is the increase in the price of drugs. This increase led to the appointment of a Departmental Committee to report upon the Drug Fund, and as a result of the report a revised plan of paying the chemists' accounts on a commercial tariff has been adopted. As a still further outcome it has been necessary to revise the regulations for the administration of medical benefit, and the new regulations came into force as from the beginning of 1916.

Closely related to medical benefit is sanatorium benefit—for they are both administered by Insurance Committees and not by approved societies. The administration of sanatorium benefit is the least satisfactory of all the benefits conferred under the National Insurance Acts. The financial basis is a grant *per capita* for each insured person within the Insurance Committee area. Owing, however, to the unequal distribution according to committee areas of insured persons suffering from tuberculosis and to differences in efficiency of administration, the anomaly has arisen that whereas in some areas full provision is made, in others there is so serious a shortage of sanatorium benefit funds that there are long waiting lists of those applying for treatment. The question requires thorough and independent inquiry, more particularly as the Treasury has withstood the grant of any special funds for those committees whose funds exhibit a shortage.

### The Demand for an Inquiry.

During the last few weeks of the year there has been evidence of uneasiness as to the financial stability of National Health Insurance. This has found expression in Parliament, where the demand has been made for an independent inquiry to be instituted. The demand appears to be supported by the vast majority of the larger approved societies, but it is resisted by the doctors and by the Government. Mr. Charles Roberts, in answering the criticisms of Mr. G. W. Currie at the beginning of December, said in Parliament that the moment was inopportune for such an inquiry in view of the fact that the staffs of the Insurance Commissioners as well as of the approved societies have been so seriously depleted through the war. It seems, therefore, that the inquiry must be deferred, but as there are good grounds for a thorough investigation of the whole of the administration, the demand must be pressed at the earliest favourable opportunity. National Health Insurance contains such vast possibilities for the future welfare of the people that it cannot be allowed to drift into insolvency or disrepute.

